# Author Correction: Cell non-autonomy amplifies disruption of neurulation by mosaic *Vangl2* deletion in mice

**DOI:** 10.1038/s41467-021-23714-8

**Published:** 2021-05-28

**Authors:** Gabriel L. Galea, Eirini Maniou, Timothy J. Edwards, Abigail R. Marshall, Ioakeim Ampartzidis, Nicholas D. E. Greene, Andrew J. Copp

**Affiliations:** 1 Developmental Biology and Cancer, UCL GOS Institute of Child Health, London, UK; 2 Comparative Bioveterinary Sciences, Royal Veterinary College, London, UK

**Keywords:** Cytoskeleton, Disease model, Morphogenesis

Correction to: *Nature Communications* 10.1038/s41467-021-21372-4, published online 19 February 2021.

The original version of this Article contained an error in the Code availability statement, which omitted a link to the in-house Fiji macro. The Code availability statement should read:

“Cellpose processing is available on GitHub https://github.com/timjedwar/Vangl2-cell-morpho.112. The in-house Fiji macro developed to identify and extract the surface of 3D structures is available from https://github.com/DaleMoulding/Fiji-Macros and related resources are available from the corresponding author on reasonable request or previously published^14^.”

This has been corrected in both the PDF and HTML versions of the Article.

