# Supplementary material for: Cell non-autonomy amplifies disruption of neurulation by mosaic Vangl2 deletion in mice
Source: Nat Commun. 2021 Feb 19;12:1159. doi: 10.1038/s41467-021-21372-4 (PMC7895924; doi:10.1038/s41467-021-21372-4)
Supplement: Supplementary file 1 — Supplementary Information [file 41467_2021_21372_MOESM1_ESM.pdf]

Cell non-autonomy amplifies disruption of neurulation by mosaic *Vangl2* deletion in mice

Gabriel L Galea[1,2], Eirini Maniou[1], Timothy J Edwards[1], Abigail R Marshall[1], Ioakeim Ampartzidis[1], Nicholas DE Greene[1], Andrew J Copp[1]

1. Developmental Biology and Cancer, UCL GOS Institute of Child Health, London, UK
2. Comparative Bioveterinary Sciences, Royal Veterinary College, London, UK

Supplementary Figure 1

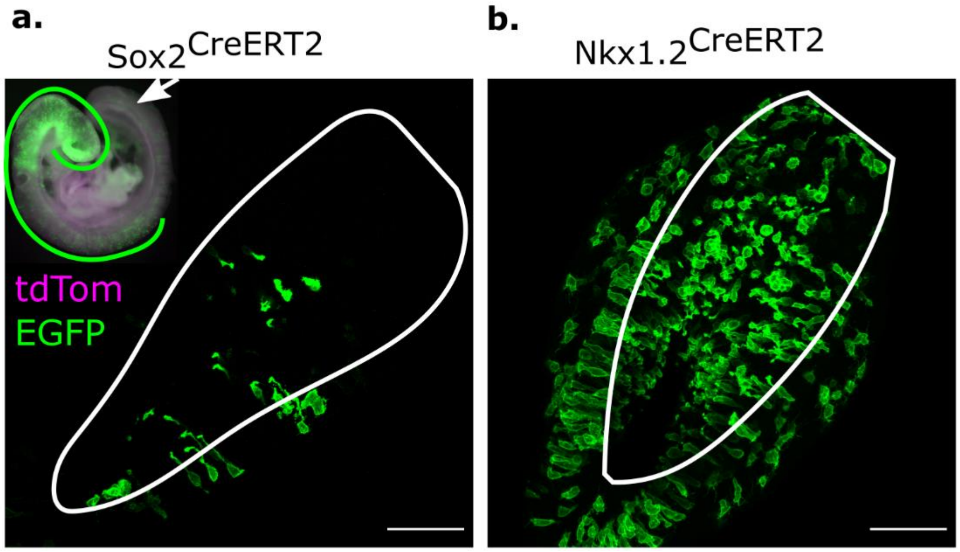

Supplementary Figure 1: Comparison of neuroepithelial lineage-tracing with different Cre drivers.

A. *Sox2*^{CreERT2} *produces robust recombination along much of the closed neural tube (green line along embryo image) but only lineage-traces a very small number of neuroepithelial cells in the open PNP (arrow) at E9.5, 24 h after tamoxifen administration.*

B. *Nkx1.2*^{CreERT2} *lineage-traces many neuroepithelial cells in the open PNP, as well as cells of the closed neural tube at E9.5, 24 hours after tamoxifen administration.*

White lines demarcate the PNP neuroepithelium. In each case recombination is assessed using the mTmG reporter construct, in which unrecombined cells express tdTomato (tdTom, not shown) and recombined cells express EGFP. CreERT2 activation was induced with tamoxifen 24 hours previously. Scale bars = 100 µm.

Supplementary Figure 2

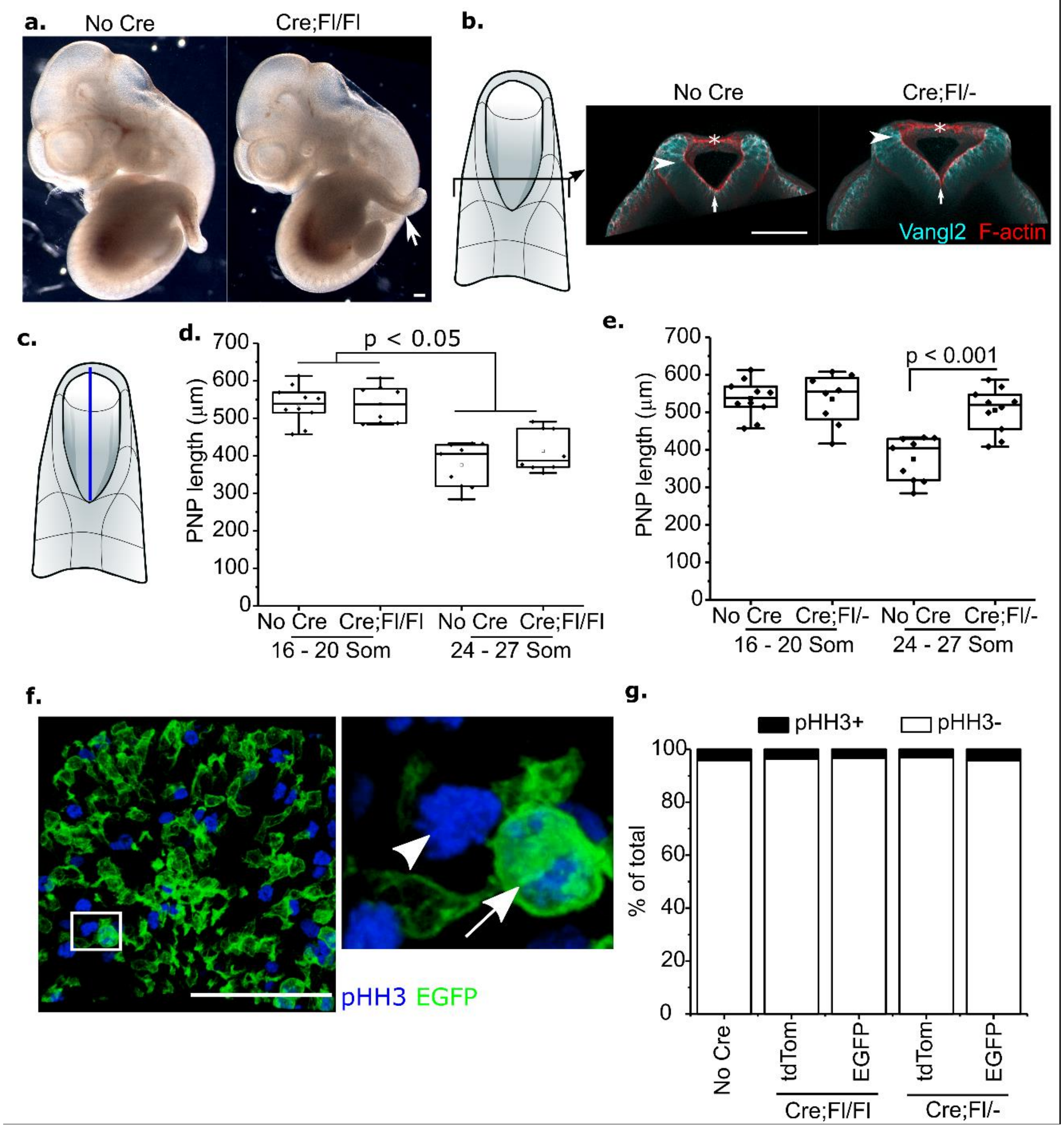

Supplementary Figure 2: Cre;Fl/Fl embryos close their PNPs but developed a dorsally curled tail.

A. *Representative images of E10.5 control (No Cre) and Cre;Fl/Fl littermate illustrating the dorsally curled tail (arrow), indicative of delayed PNP closure in some Cre;Fl/Fl embryos.*

B. *3D reconstructions of a No Cre and Cre;Fl/- PNP from embryos collected at E9.5 showing the dorsolateral hinge points (arrowhead) and medial hinge point (arrow). * denotes zippering point.*

C. *Illustration of PNP length quantification (blue line).*

D-E. *Quantification of PNP length in control versus D: Cre;Fl/Fl and E: Cre;Fl/- embryos at the indicated somite stages (E9-E9.5). PNP length decreases in both genotypes. * $p < 0.05$ by ANOVA with Bonferroni post-hoc. 16-20 Som No Cre = 10, Cre;Fl/Fl = 8, Cre;Fl/- = 8 embryos. 24-27 Som No Cre = 9, Cre;Fl/Fl = 8, Cre;Fl/- = 10 embryos.*

F. *3D reconstruction of a Cre;Fl/- PNP showing EGFP and pHH3 wholemount staining. Mitotic (pHH3+) nuclei are present in both recombined (arrow) and non-recombined (arrowhead) cells.*

G. *Proportion of cells stained positive for pHH3 is comparable in control, tdTom+ and EGFP+ cells, indicating that mitosis is unaffected by Vangl2 recombination. No Cre 6908 cells from 6 embryos. Cre;Fl/Fl EGFP/tdTom 1172/7754 cells from 8 embryos. Cre;Fl/- EGFP/tdTom 1046/6702 cells from 7 embryos.*

Scale bars = 100 µm.

Supplementary Figure 3

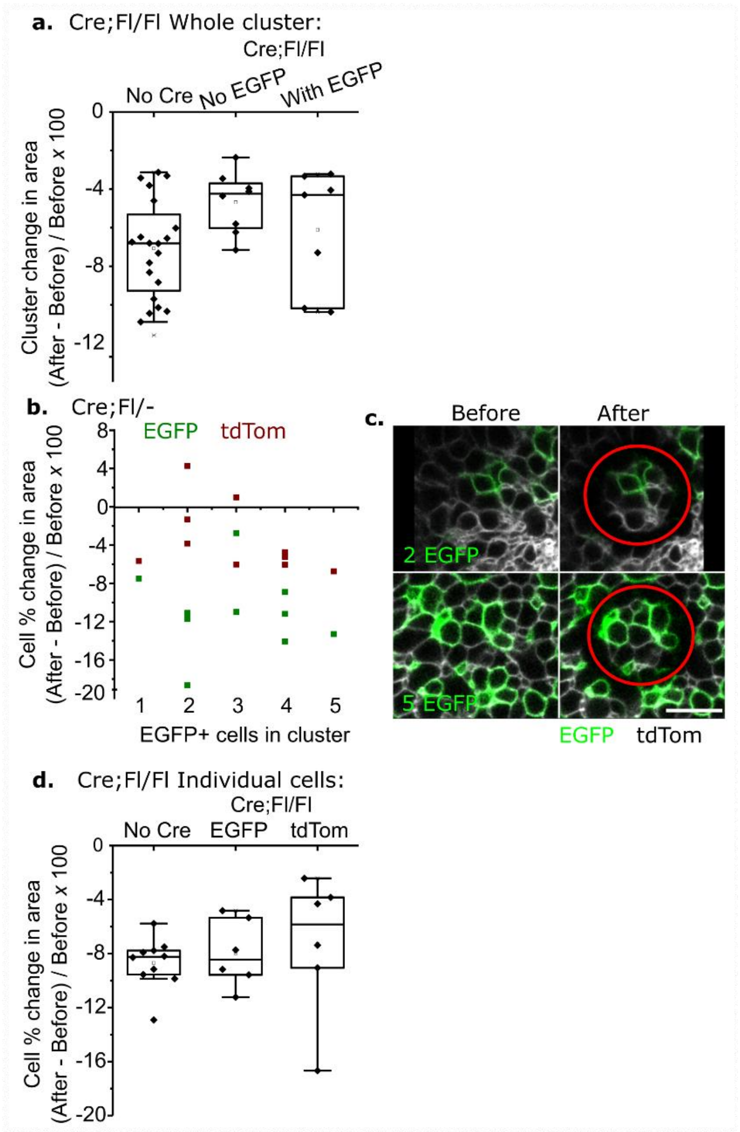

Supplementary Figure 3: Neuroepithelial apical retraction after annular ablation is unaffected in Cre;Fl/Fl embryos.

A. *Quantification of the retraction (% change in area) of cell clusters within the ablated annulus. Dots represent a cluster in an individual embryo; each embryo was ablated once only. Control embryos are compared to Cre;Fl/Fl embryos. No Cre = 20, No EGFP = 8, With EGFP = 7 embryos.*

B. *Quantification of the retraction (% change in area) of EGFP+ and tdTom+ cells within the ablated annulus of Cre;Fl/- embryos graphed against the number of EGFP+ cells contacting the annulus. EGFP+ cells were counted even if they were themselves cut during ablation and not part of the analysed cluster. Each point represents EGFP+ or tdTom+ cells from an individual embryos, n = 10.*

C. *Representative ablations showing clusters with two and five EGFP cells. Scale = 20 µm.*

D. *Quantification of the retraction (% change in area) of individual cells within the ablated annulus. No Cre = 10, Cre;Fl/Fl = 6 embryos.*

Control embryos are the same as in Figure 3. Points represent individual embryos.

Supplementary Figure 4

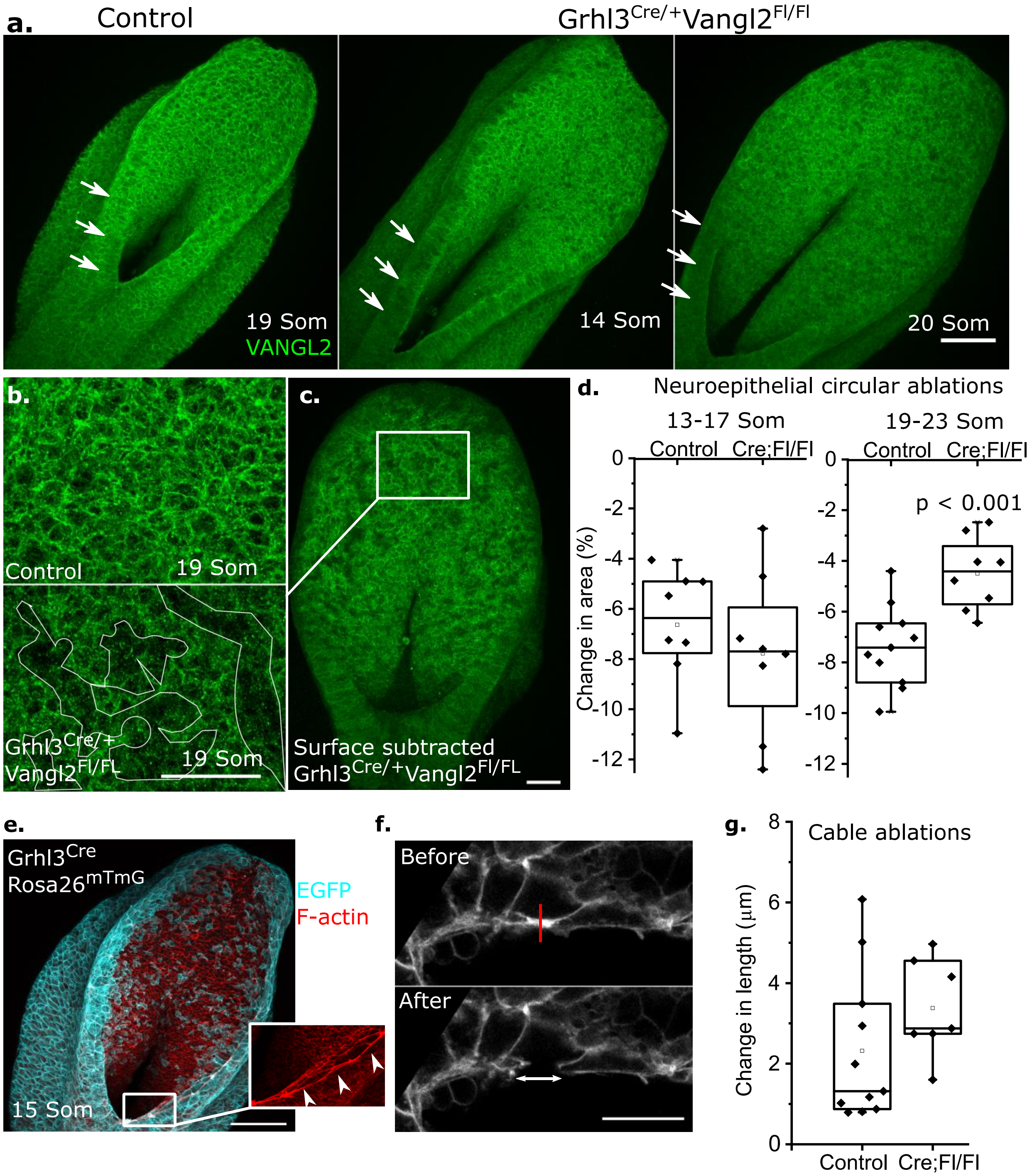

Supplementary Figure 4: Apical neuroepithelial tension is specifically diminished by mosaic Vangl2 deletion using Grhl3^{Cre}. All panels in this figure show data using the non-inducible *Grhl3*^{Cre}, not *Nkx1.2*^{CreERT2} used throughout the remainder of the manuscript.

A. *As previously reported*[1]*, Grhl3*^{Cre} *produces loss of VANGL2 protein in surface ectoderm (arrows) early in development, but neuroepithelial VANGL2 is only diminished at later stages. Scale bar = 100 µm.*
B. *Surface-subtracted images of somite stage-matched control and Grhl3*$^{Cre/+}$*Vangl2*$^{Fl/Fl}$ *embryos showing a mosaic pattern of VANGL2 loss in the apical neuroepithelium of the latter. Scale bar = 50 µm.*
C. *Low magnification image to show the location of the neuroepithelial cells in B. Scale bar = 50 µm.*
D. *Quantification of the change in cluster area following annular laser ablation of the apical neuroepithelium. Ablation experiments were carried out at two developmental stages: 13-17 somite stages before VANGL2 protein depletion is apparent and 19-23 somite stage after loss of VANGL2 becomes visible. Cluster retraction is equivalent between controls at both stages of development and is selectively diminished in embryos which have lost VANGL2 (p = 0.0008 by two-sided T-Test). Ablations are assumed to include Vangl2-deleted cells (the vast majority of ablation annuli would include at least one lineage-traced cell). Points represent individual embryos: 13-17 Som Control = 8 and Cre;Fl/Fl = 8, 19-23 Som Control = 11 and Cre;Fl/Fl = 8 embryos.*
E. *Representative Grhl3*^{Cre} *lineage tracing using the mTmG reporter showing near-ubiquitous recombination in the surface ectoderm which assembles long F-actin cables (arrows in inset). Also note the mosaic recombination scattered throughout the neuroepithelium at the 15 somite stage. Scale bar = 100 µm.*
F. *Representative ablation of the cell borders which assemble the F-actin cables showing recoil perpendicular to the ablation (red line). Scale bar = 20 µm.*
G. *Quantification of the change in cell border length immediately following cable ablation of 20-26 somite stage control (n = 11) and littermate Grhl3*$^{Cre/+}$*Vangl2*$^{Fl/Fl}$ *(n = 7) embryos. Points represent individual embryos. There is no significant difference in length change between genotypes by T-test.*

Supplementary Figure 5

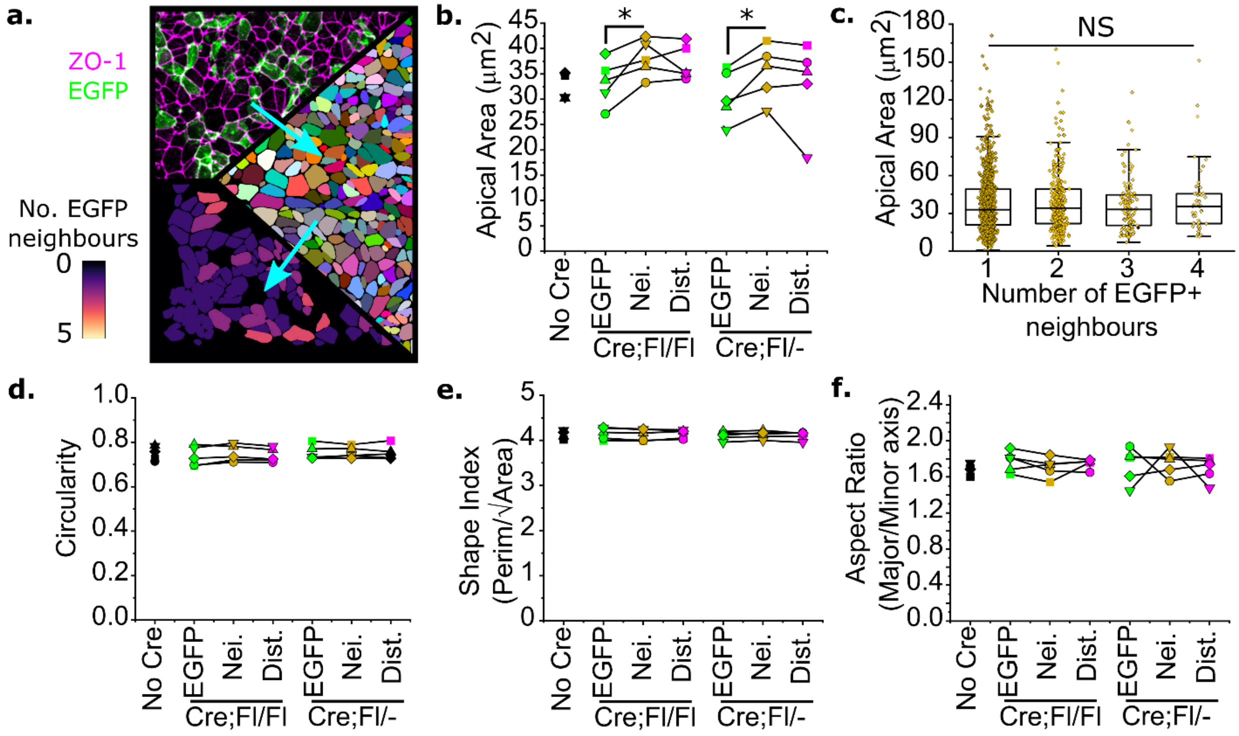

Supplementary Figure 5: *Vangl2*-deleted cells have more constricted apical areas than their *Vangl2*-replete neighbouring cells.

A. *Schematic representation of the automated image segmentation process using Cellpose to identify cell borders, attribute EGFP status based on intensity cut-offs (see Methods) and identify the number of EGFP+ cells each cell contacts.*

B. *Quantification of apical areas between the cell types indicated, showing EGFP+ cells (Vangl2-deleted) are significantly smaller than their fully Vangl2-replete neighbours in both the Cre;Fl/Fl and Cre;Fl/- embryos analysed. Points represent average values for cell types from individual embryos, lines link cell types from the same embryo. * $p < 0.05$ by mixed model analysis accounting for repeated measures from the same embryos, n = 5 embryos per genotype.*

C. *Apical area of individual Vangl2-replete cells contacting single or multiple Vangl2-deleted (EGFP) neighbours. Points represent individual cells from five embryos. All p values > 0.05 by mixed model analysis accounting for repeated measures from the same embryos; 967 cells with 1 partner, 332 with 2, 122 with 3 and 53 with 4.*

D-F. *Comparison of the standard morphometric parameters:* ***D)*** *circularity,* ***E)*** *shape index and* ***F)*** *aspect ratio, showing no significant differences in these parameters between apical surfaces of the cell types analysed. n = 5 embryos per genotype.*

Supplementary Figure 6

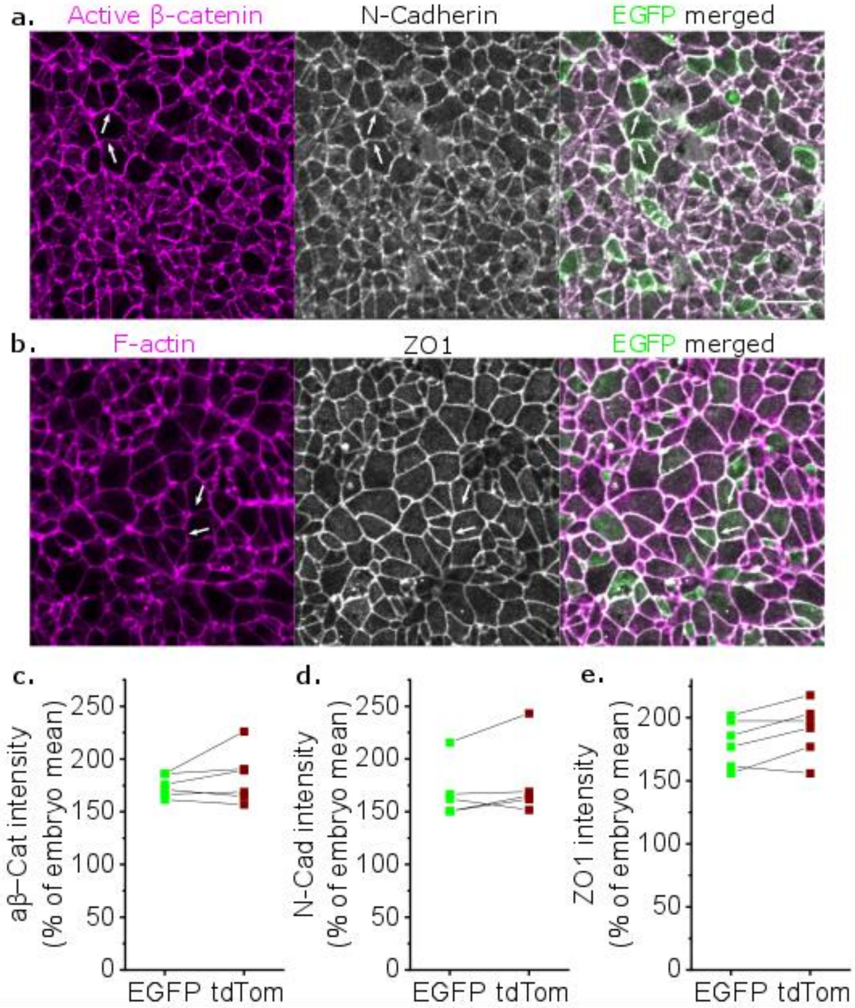

Supplementary Figure 6: *Vangl2*-deleted cells retain adherens and tight junctions.

A,B. *Surface-subtracted neuroepithelium of Cre;Fl/- embryos wholemount stained with the adherens junction markers active (de-phosphorylated) β-catenin and N-cadherin (A), phalloidin (F-actin) and the tight junction marker ZO1 (B). Arrows indicate borders between adjacent EGFP+ (Vangl2-deleted) cells shown in the merged images. Scale bars = 20 μm.*

C-E. *Quantification of each of the markers indicated in Vangl2-deleted (EGFP) and Vangl2-replete (tdTom) cells. Each point represents the average of an individual embryo (C. n = 6, D. n =5, E. n= 6) and lines join points from the same embryo. None were significantly different between Vangl2-deleted versus replete cells.*

Supplementary Figure 7

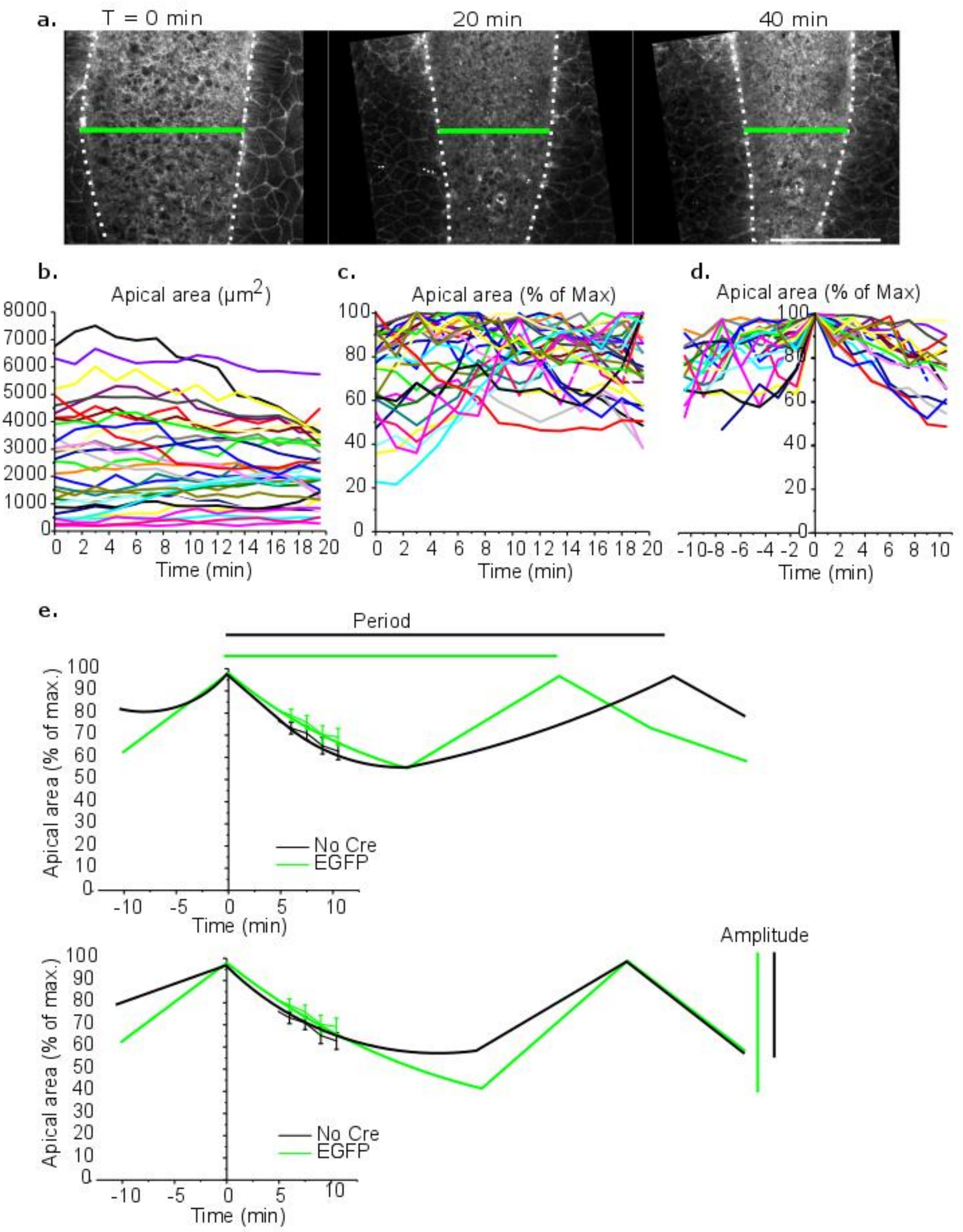

Supplementary Figure 7: Analysis of mouse neuroepithelial live imaging.

A. *Live-imaging of a mouse PNP over 40 minutes showing progression of narrowing (green line). Scale = 100 μm.*

B-D. *Processing of apical area data generated from a pilot live-imaging experiment. Each trace represents an individual cell.* ***B:*** *Raw data illustrating the wide range of initial apical areas, with some cells constricting while others dilate.* ***C:*** *Normalisation of apical area traces to the maximum observed size for each individual cell. Individual cells reach their maximum apical area randomly throughout the live imaging period.* ***D:*** *Registration of normalised live-cell imaging traces such that their maximum observed apical area is at T = 0 min.*

E. *Potential explanations for the data in Figure 4C. Vangl2-deletion could cell-autonomously decrease the period of apical constriction pulses (top panel) and/or increase pulse amplitude (bottom panel). Points represent the mean ± SEM.*

Supplementary Figure 8

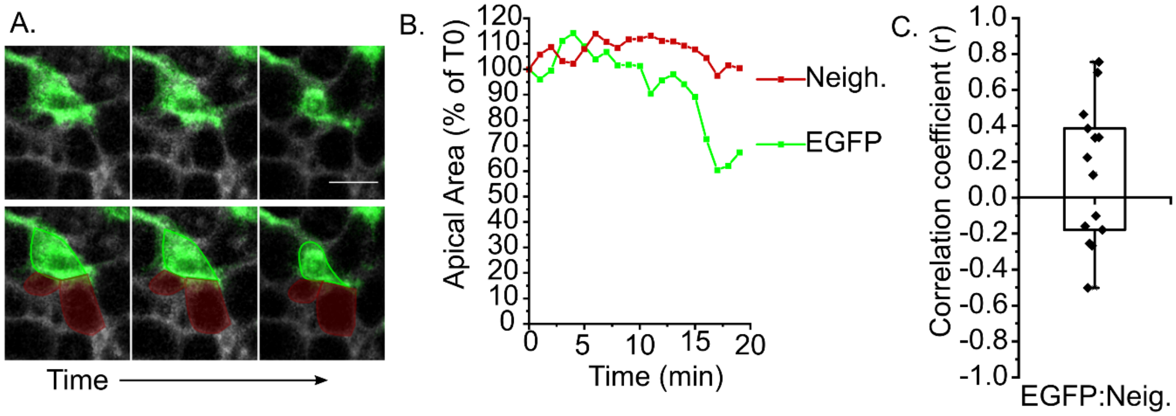

Supplementary Figure 8: Changes in apical areas of live-imaged *Vangl2*-deleted cells are not correlated with that of their *Vangl2*-replete neighbours.

A. *Live-imaged apical surface of a Cre;Fl/- embryo showing constriction of the Vangl2-deleted cell (green) without substantial change in the apical area of its Vangl2-replete neighbours (grey outlines, illustrated in maroon in the bottom panel). Panels ~5 minutes apart, scale bar = 10 µm.*

B. *Quantification of apical area in a representative Vangl2-deleted (EGFP) and neighbouring Vangl2-replete pair over time.*

C. *Correlation coefficient of changes in apical areas over time (as in B) from 30 EGFP-Neighbour cell pairs from 3 independent embryos, showing generally weak correlation.*

Supplementary Figure 9

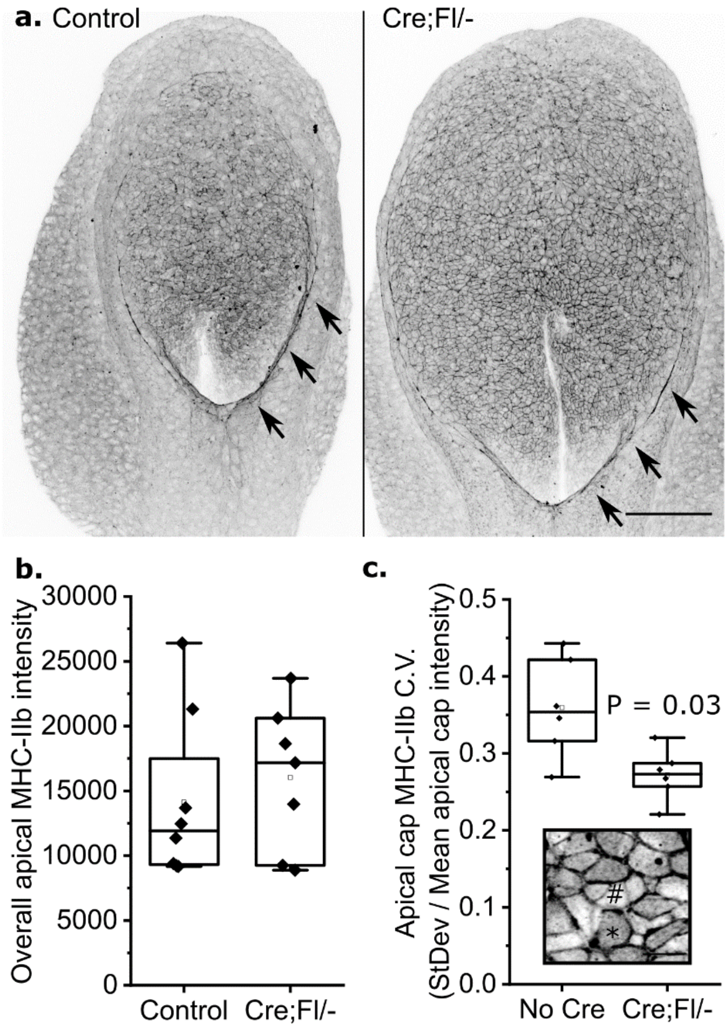

Supplementary Figure 9: Mosaic Vangl2 deletion does not significantly alter apical neuroepithelial MHC-IIb intensity, but reduces its variability between cells on the apical cap.

A. *Maximum-projected images of a control and littermate Cre;Fl/- embryo, each at the 23 somite stage, wholemount stained to show MHC-IIb. Arrows indicate the neural fold F-actin cables, scale bar = 100 µm.*

B. *Quantification of overall MHC-IIb staining intensity, which is not significantly different between control (n = 8) and Cre;Fl/- (n = 7) embryos. Points represent individual embryos.*

C. *The coefficient of variation (C.V.) of MHC-IIb apical cap intensity was quantified in control (n = 6) and Cre;Fl/- (n = 6) embryos as a measure of variability in apical cap myosin intensity. This variability results from cells having low (#) or high (*) apical cap staining, as shown in the inset. P value by two-sided T-test. Scale = 10 µm.*

Supplementary Figure 10

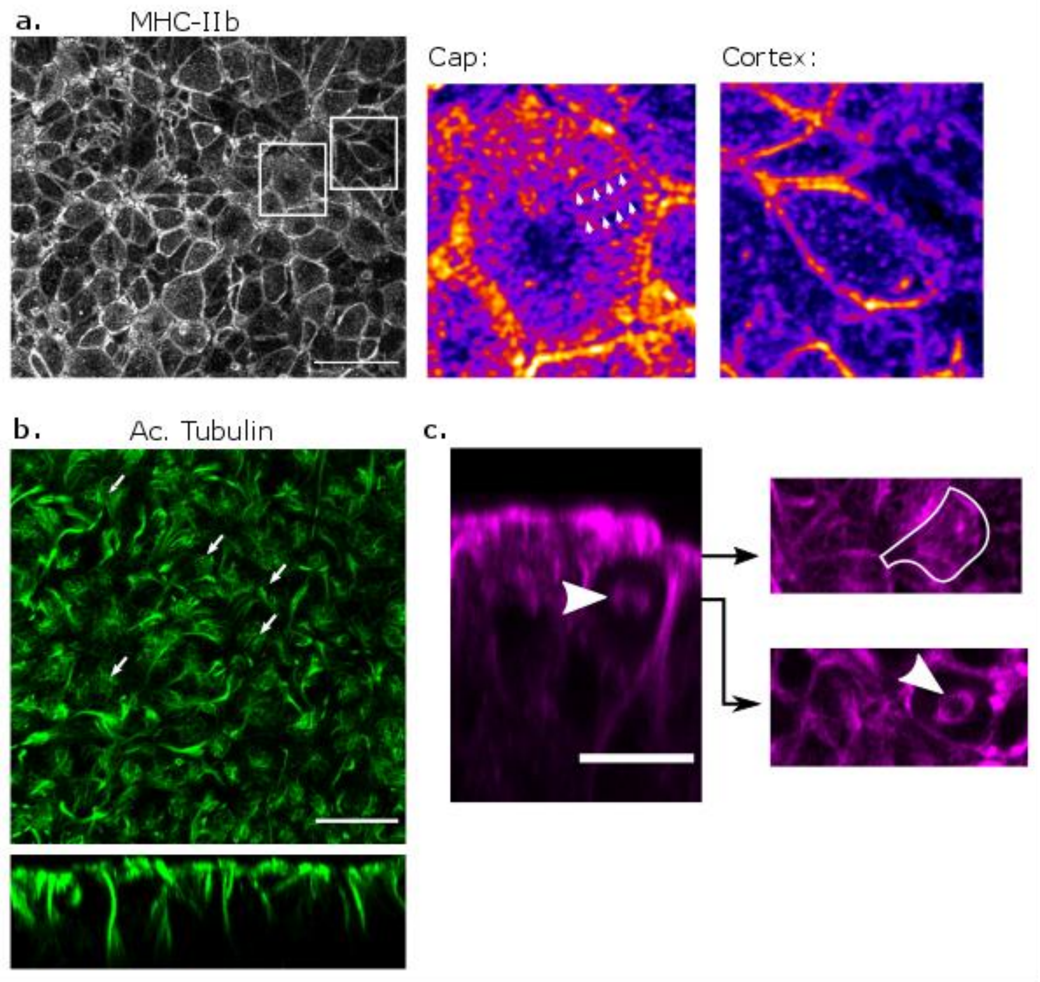

Supplementary Figure 10: Neuroepithelial MHC-IIb and acetylated tubulin staining.

A. *MHC-IIb (Abcam antibody) staining showing neuroepithelial apical cap and cortical staining. White boxes indicate the cells with apical cap or cortex staining shown in fire LUT. Arrows indicate apical cap arrangements.*

B. *Acetylated tubulin staining showing labelling of both neuroepithelial apical "radial" organisations (arrows) and apico-basal "tails" (bottom optical cross-section).*

C. *Optical cross sections through tubulin-stained neuroepithelium showing the distinct apical (white outline) and centrosomal (arrowhead) microtubule networks.*

Scale bars = 20 µm.

Supplementary References

1 Galea, G. L. *et al.* Vangl2 disruption alters the biomechanics of late spinal neurulation leading to spina bifida in mouse embryos. *Dis Model Mech* **11**, doi:10.1242/dmm.032219 (2018).
