# Supplementary material for: Cell non-autonomy amplifies disruption of neurulation by mosaic Vangl2 deletion in mice
Source: Nat Commun. 2021 Feb 19;12:1159. doi: 10.1038/s41467-021-21372-4 (PMC7895924; doi:10.1038/s41467-021-21372-4)
Supplement: Supplementary file 3 — Reporting Summary [file 41467_2021_21372_MOESM3_ESM.pdf]

nature research

Corresponding author(s): *Double-blind peer review submissions: write DBPR and your manuscript number here instead of author names.*

Last updated by author(s): *YYYY-MM-DD*

Reporting Summary

Nature Research wishes to improve the reproducibility of the work that we publish. This form provides structure for consistency and transparency in reporting. For further information on Nature Research policies, see our Editorial Policies and the Editorial Policy Checklist.

Statistics

For all statistical analyses, confirm that the following items are present in the figure legend, table legend, main text, or Methods section.

n/a	Confirmed	
☐	☒	The exact sample size (*n*) for each experimental group/condition, given as a discrete number and unit of measurement
☐	☒	A statement on whether measurements were taken from distinct samples or whether the same sample was measured repeatedly
☐	☒	The statistical test(s) used AND whether they are one- or two-sided<br>*Only common tests should be described solely by name; describe more complex techniques in the Methods section.*
☐	☒	A description of all covariates tested
☐	☒	A description of any assumptions or corrections, such as tests of normality and adjustment for multiple comparisons
☐	☒	A full description of the statistical parameters including central tendency (e.g. means) or other basic estimates (e.g. regression coefficient) AND variation (e.g. standard deviation) or associated estimates of uncertainty (e.g. confidence intervals)
☐	☒	For null hypothesis testing, the test statistic (e.g. *F*, *t*, *r*) with confidence intervals, effect sizes, degrees of freedom and *P* value noted<br>*Give P values as exact values whenever suitable.*
☒	☐	For Bayesian analysis, information on the choice of priors and Markov chain Monte Carlo settings
☒	☐	For hierarchical and complex designs, identification of the appropriate level for tests and full reporting of outcomes
☐	☒	Estimates of effect sizes (e.g. Cohen's *d*, Pearson's *r*), indicating how they were calculated

Our web collection on statistics for biologists contains articles on many of the points above.

Software and code

Policy information about availability of computer code

Data collection	Zeiss Zen Blue and Zen Black
Data analysis	Fiji/Image J<br>Origin Pro<br>Cellpose: https://github.com/timjedwar/Vangl2-cell-morpho

For manuscripts utilizing custom algorithms or software that are central to the research but not yet described in published literature, software must be made available to editors and reviewers. We strongly encourage code deposition in a community repository (e.g. GitHub). See the Nature Research guidelines for submitting code & software for further information.

Data

Policy information about availability of data

All manuscripts must include a data availability statement. This statement should provide the following information, where applicable:

- Accession codes, unique identifiers, or web links for publicly available datasets
- A list of figures that have associated raw data
- A description of any restrictions on data availability

Values used to generate each graph are provided in the extended data. All reagents are commercially available. In-house Fiji processing tools and related resources are available from the corresponding author on reasonable request.

Field-specific reporting

Please select the one below that is the best fit for your research. If you are not sure, read the appropriate sections before making your selection.

- [x] Life sciences
- [ ] Behavioural & social sciences
- [ ] Ecological, evolutionary & environmental sciences

For a reference copy of the document with all sections, see nature.com/documents/nr-reporting-summary-flat.pdf

Life sciences study design

All studies must disclose on these points even when the disclosure is negative.

Sample size	Page 20: Sample sizes for morphometric and laser ablation experiments were based on previous studies.<br>Page 9: A pilot study analysing a wild-type embryo showed 24 cells need to be analysed to detect a 20% difference in apical area (p = 0.05, power = 0.8).<br>Page 20: Sample sizes for morphometric and laser ablation experiments were based on previous studies. A pilot study of quantifying apical constriction is described in the results.
Data exclusions	Page 20: Thus, analyses were generally blinded to Vangl2 deletion status and no embryos were excluded after analysis.
Replication	Methods:<br>All images are representative of observations in at least three embryos from independent litters.
Randomization	Not applicable as all embryos at the relevant somite stages were analysed.
Blinding	Page 20: Blinding to CreERT2 positivity was generally not possible, but analyses were carried out without knowing whether embryos were Cre;Fl/Fl (no phenotype) or Cre;Fl/-. Thus, analyses were generally blinded to Vangl2 deletion status and no embryos were excluded after analysis. There were three exceptions to this blinding. The first exception is when Vangl2 itself was visualized given loss of Vangl2 signal was obvious in Cre;Fl/- embryos only; this data is analyzed quantitatively. The second exception was when selecting embryos for live imaging. In order to ensure that control and experimental embryos were imaged, GLG inspected neural fold eversion, which by then was a recognized feature of Cre;Fl/- embryos (note that this is only possible when the whole PNP can be seen, not when processing zoomed images of the apical neuroepithelium). This was only miss-judged in one embryo. The third exception was AiryScan imaging of myosin and tubulin, for which only experimental embryos could be imaged due to processing limitations. To circumvent this, when quantifying tubulin tail length each tail was saved as a separate image with a blinded key indicating whether it was EGFP+ or not.

Reporting for specific materials, systems and methods

We require information from authors about some types of materials, experimental systems and methods used in many studies. Here, indicate whether each material, system or method listed is relevant to your study. If you are not sure if a list item applies to your research, read the appropriate section before selecting a response.

Materials & experimental systems

n/a	Involved in the study
[ ]	[x] Antibodies
[x]	[ ] Eukaryotic cell lines
[x]	[ ] Palaeontology and archaeology
[ ]	[x] Animals and other organisms
[x]	[ ] Human research participants
[x]	[ ] Clinical data
[x]	[ ] Dual use research of concern

Methods

n/a	Involved in the study
[x]	[ ] ChIP-seq
[x]	[ ] Flow cytometry
[x]	[ ] MRI-based neuroimaging

Antibodies

Antibodies used	Primary antibodies were: rabbit anti-VANGL2 (Millipore clone 2G4, as previously validated74, 1:100 dilution), rabbit anti-MHCIIb (BioLegend PRB-445 and Abcam clone 3H2, 1;200), rabbit anti-Ser19 pMLCII (Cell Signalling Technology #3671, 1:100), rabbit anti-K40 acetylated α-TUBULIN (Abcam EPR16772, 1:200), mouse anti-β TUBULIN (Insight Biotechnology clone AT5B2, 1:200), mouse anti-β-CATENIN (Santa Cruz Biotechnology, clone E-5, 1:100), mouse anti-N-cadherin (Cell Signalling Technology clone 13A9, 1:150), rabbit anti-ZO1 (Thermo Scientific clone 40-2200, 1:100), rabbit anti-ROCK1 (Abcam, ab45171, 1:100) and chicken anti-GFP (Abcam ab13970, 1:300).

Validation	All are commercially available antibodies validated by the producer.<br>The Vangl2 2G4 antibody was additionally knockout validated in this study and a previous study from our group.<br>Two MHC-IIb antibodies were used to validate the localisation of their target.<br>Inhibition of Rho/ROCK signalling by the compound Y27632 was previously reported by our group (Escuin et al, J Cell Sci, 2015)

Animals and other organisms

Policy information about studies involving animals; ARRIVE guidelines recommended for reporting animal research

Laboratory animals	Methods: All mouse colonies were maintained in house on a C57Bl/6 background and bred from 8 weeks of age.
Wild animals	NA
Field-collected samples	NA
Ethics oversight	Studies were performed under the UK Animals (Scientific Procedures) Act 1986 and the Medical Research Council's Responsibility in the Use of Animals for Medical Research (1993).

Note that full information on the approval of the study protocol must also be provided in the manuscript.
